# Safety findings from CENTURION, a phase 3 consistency study of lasmiditan for the acute treatment of migraine

**DOI:** 10.1186/s10194-021-01343-2

**Published:** 2021-11-06

**Authors:** C Tassorelli, S Bragg, JH Krege, EG Doty, PA Ardayfio, D Ruff, SA Dowsett, T Schwedt

**Affiliations:** 1 University of Pavia, Pavia, Lombardy Italy; 2 Eli Lilly and Company, IN 46285 Indianapolis, USA; 3 Mayo Clinic, Phoenix, AZ USA

**Keywords:** Lasmiditan, Clinical trial, Migraine, Safety, Adverse events, Multiple attacks, Consistency

## Abstract

**Background:**

Lasmiditan (LTN) is a selective 5-HT_1F_ receptor agonist for the acute treatment of migraine in adults. We present detailed safety findings from the placebo-controlled, double-blind Phase 3 study, of LTN treatment across 4 attacks (CENTURION).

**Methods:**

Patients were randomized 1:1:1 to LTN 200 mg (LTN200), LTN100, or a control group that received placebo for 3 attacks and LTN50 for either the 3rd or 4th attack (1:1). Safety analyses were conducted for patients who took ≥1 dose of study drug and, in some cases, those who took all 4 doses.

**Results:**

Overall, 1471 patients treated 4494 attacks. The incidences of treatment-emergent serious adverse events (SAEs) were - placebo, *n*=2 (0.4 %); LTN100, *n*=1 (0.2 %); LTN200, *n*=2 (0.4 %); no specific treatment-emergent SAE was reported in more than one patient. The most common treatment emergent adverse events (TEAEs) with lasmiditan were dizziness, paresthesia, fatigue, nausea, vertigo, and somnolence; the vast majority were mild or moderate in severity. The incidences of these TEAEs were highest during the first attack and decreased during subsequent attacks. For patients who experienced a common TEAE with the first attack, less than 45 % experienced the same event in subsequent attacks. Patients who did not experience an event in the 1st attack infrequently experienced the same event in subsequent attacks.

The time of onset of the common TEAE ranged from ~40 min to 1 h (dependent upon TEAE) and, for individual TEAE, the onset was similar across attacks. Duration was dependent upon TEAE and attack. It was shortest for paresthesia (< 2 h for all attacks); it ranged from 1.8 to 5.5 h for other common TEAEs and was generally similar across attacks.

Serotonin syndrome was reported for 2 patients post LTN dosing; there were no meaningful differences across treatment groups in suicidality; there was no evidence of an increase in motor vehicle accidents.

**Conclusion:**

In this blinded, controlled, multiple-attack study, LTN was associated with generally mild or moderate CNS-related TEAEs of short duration. TEAEs tended to decrease in frequency across the 4 attacks.

**Trial registration:**

NCT03670810

**Supplementary Information:**

The online version contains supplementary material available at 10.1186/s10194-021-01343-2.

## Introduction

The value of an acute treatment for migraine depends not only on its ability to rapidly and consistently relieve symptoms, but also on acceptable drug safety and tolerability.

Lasmiditan is a selective serotonin (5-HT_1F_) receptor agonist (ditan), approved in the US [1] and the United Arab Emirates for the acute treatment of migraine with or without aura in adults and being investigated in other geographies. In Phase 3 studies, lasmiditan was effective in the treatment of a migraine attack, as measured by pain freedom, pain relief, most bothersome symptom (MBS) freedom at 2 h, [2–4] and demonstrated a consistent response across attacks [4]. Common treatment emergent adverse events (TEAEs) were dizziness, paresthesia, somnolence, fatigue, nausea, muscular weakness, and hypoesthesia [4, 5].

Here we report the detailed safety findings from the CENTURION study, a large Phase 3 placebo-controlled study designed to assess the efficacy, consistency, and safety of lasmiditan in acute treatment of 4 migraine attacks with or without aura.

## Methods

CENTURION was a multicenter, placebo-controlled, double-blind Phase 3 study conducted in Europe, North America and Asia. The study was designed to assess the efficacy, consistency of response, and safety of lasmiditan in the acute treatment of 4 migraine attacks with or without aura. Details of the study design have been published elsewhere [4].

Patients were randomized in a blinded fashion in a 1:1:1 ratio to one of 3 treatment groups (1) lasmiditan 200 mg for 4 attacks; (2) lasmiditan 100 mg for 4 attacks; or (3) a control group that received placebo for 3 attacks and lasmiditan 50 mg for 1 attack. The control group comprised 2 treatment sequences (1:1) whereby one group received lasmiditan 50 mg for the 3rd attack and the other received lasmiditan 50 mg for the 4th attack (Table 1). The inclusion of the control group provided the following: the opportunity to compare the efficacy and consistency of response to lasmiditan 100 mg and 200 mg to placebo; improved maintenance of blinding, since patients in the control group received lasmiditan 50 mg for 1 attack; more patient acceptability since they did not receive placebo for all attacks; and the opportunity to assess the efficacy of lasmiditan 50 mg during 1 attack. Participants were not to be informed about the randomization scheme; instead, they were to be informed that no participant would receive placebo for all attacks.

**Table 1:** Randomization in CENTURION

Randomized 1:1:1↓	Attack 1	Attack 2	Attack 3	Attack 4
LTN 100 (*N*=539)	**LTN 100**	**LTN 100**	**LTN 100**	**LTN 100**
LTN 200 (*N*=536)	**LTN 200**	**LTN 200**	**LTN 200**	**LTN 200**
Control (*N*=538) *Randomized 1:1*→	PBO	PBO	**LTN 50**	PBO
	PBO	PBO	PBO	**LTN 50**

Participants were asked to treat 4, preferably consecutive, migraine attacks with the study drug. The treatment period concluded after 4 treated migraine attacks or 4 months after randomization, whichever came sooner. Phone visits were conducted after the 1st attack or at 1 month after randomization, whichever came sooner, and at 3 months after randomization. A site visit occurred at 2 months after randomization.

### Tolerability and safety

Adverse events and symptoms between migraine attacks as well as any unusual symptoms within 48 h after dosing were to be recorded by patients in a paper journal. Sites were to use information from the paper journal to help define and record AE information.

Tolerability and safety evaluation included the assessment of TEAEs, defined as new or worsening adverse events during the 48 h after a dose of study drug.

Severity of TEAEs was rated by the investigator based on patient feedback. Events that were easily tolerated were considered mild severity; moderate severity implied enough discomfort with the event to interfere with usual activity; severe events were incapacitating with inability to work or perform usual activity.

Suicidal ideation and behaviors were assessed with the use of the Columbia–Suicide Severity Rating Scale (C-SSRS) [6]. A driving questionnaire was administered to assess for motor vehicle accidents or moving violations/citations after taking study drug.

### Statistical analysis

Summaries of adverse events were performed using all patients who treated at least one attack as well as using only patients who treated 4 attacks during the double-blind period. Additionally, summaries were also performed within geographical regions. For summaries of common TEAEs by severity, if the patient experienced the same event multiple times during the same attack, the severity for that event was defined as that of worst severity. For a given common TEAE, the onset was defined as the time from dosing until the reported start time of the event. In the situation where multiple cases of the same event occurred with a given treated attack, the time to onset was defined as the time to dosing until the start of the first case. Similarly, event duration for a given event was defined as the time from the start of the first reported case for a given attack until the last reported ending time for the same event/attack. Events with missing start/stop times were not included in calculations of onset or duration. No formal statistical hypothesis tests were performed.

## Results

of the 1613 patients randomized in CENTURION, 1471 (91 %) treated 1 or more migraine attacks with study drug and were considered the safety population; approximately half of those randomized (49 %) treated all 4 migraine attacks. Overall, 4494 attacks were treated. Further details of drug exposure are shown in Table S1.

Demographics have been previously published [4]. Briefly, the mean age was 42 years, 84 % were female. The majority of trial participants (76 %) were from Europe, 12 % from North America, and 12 % from Asia.

Over the course of the study, patients treated ≤ 4 attacks with lasmiditan 100 mg, ≤4 attacks with lasmiditan 200 mg or ≤ 3 attacks with placebo *and* ≤1 with lasmiditan 50 mg.

### Adverse event summary

There were no deaths in the study and the incidence of serious AEs across attacks was similar in the lasmiditan 100 mg, lasmiditan 200 mg and placebo treatment groups (Table 2). Of the serious AEs, 5 were considered treatment emergent (placebo (0.4 %) - liver disorder, suicidal ideation; lasmiditan 100 mg (0.2 %) - asthma; lasmiditan 200 mg (0.4 %)- hemiplegic migraine and serotonin syndrome). There were no serious AEs reported after dosing with lasmiditan 50 mg group (Table 3).

**Table 2:** Adverse event summary over the course of the study (safety population)

	Placebo (*N*=500)	Lasmiditan 100 mg (*N*=485)	Lasmiditan 200 mg (*N*=486)
**Across Attacks**:			
*Attacks treated*	*≤3*	*≤4*	*≤4*
Deaths	0	0	0
Serious AE^a^, n (%)	7 (1.4)	7 (1.4)	8 (1.6)
Study discontinuation due to AE^b^, n (%)	6 (1.2)	36 (7.4)	38 (7.8)
**1st attack only**:			
% reporting ≥ 1 TEAE	22.4	53.0	61.1
Most common TEAEs			
- Dizziness	23 (4.6)	108 (22.3)	129 (26.5)
- Paresthesia	9 (1.8)	39 (8.0)	62 (12.8)
- Fatigue	9 (1.8)	37 (7.6)	46 (9.5)
- Nausea	19 (3.8)	31 (6.4)	49 (10.1)
- Vertigo	1 (0.2)	24 (4.9)	33 (6.8)
- Somnolence	7 (1.4)	20 (4.1)	37 (7.6)

*Abbreviations* : *AE* adverse event, *TEAE* treatment-emergent AE

^a^Of the serious AEs, 5 were considered treatment emergent: Placebo =2 (liver disorder, suicidal ideation); lasmiditan 100 mg=1 (asthma); lasmiditan 200 mg=2 (hemiplegic migraine [judged unrelated to study drug by investigator] and serotonin syndrome [met Sternbach’s and Hunter’s criteria] )

^b^Dizziness most common TEAE leading to discontinuation

**Table 3:** Adverse event summary for the control group in Attacks 3 and 4 (safety population)

	Control Group (*N*=266)^a^	
	**Placebo**	**Lasmiditan 50mg**^b^
*Attacks treated*	*3rd or 4th*	*3rd or 4th*
% reporting common TEAE^c^		
- Dizziness	2.6	5.6
- Paresthesia	1.5	1.1
- Fatigue	0.8	2.3

*Abbreviations* : *AE* adverse event, *TEAE* treatment-emergent AE

^a^For patients who treated 4 attacks

^b^No deaths, serious AEs, or AEs leading to discontinuation with lasmiditan 50 mg

^c^Common TEAE – reported in >2 % of LTN group

Overall, 7 to 8 % in the lasmiditan 100 mg and lasmiditan 200 mg treatment groups discontinued due to an AE (Table 2), no patients discontinued after taking lasmiditan 50 mg in the 3rd or 4th attack (Table 3), and 1.2 % discontinued after taking placebo (all in the 1st or 2nd attack).

### Common TEAEs - detailed findings

The most common TEAEs with lasmiditan 100 mg or 200 mg in the first attack were dizziness, nausea, paresthesia, fatigue, somnolence, and vertigo (Table 2).

Examples of reported terms of dizziness included “dizziness,” “giddiness,” “lightheadedness,” “feeling faint,” “intermittent dizziness,” and “woozy feeling.” Examples of reported terms for paraesthesia included “paraesthesia,” “tingling sensation,” and “pins and needles”; these sensations were described as generalized (entire body) or in specific locations such as face, tongue, jaw, arms, hands, fingertips, legs, or feet. Examples of reported terms for vertigo included “vertigo,” “staggering vertigo,” “dizziness (spinning sensation consistent with vertigo),” “dizziness (vertigo),” “recurrent vertigo,” “whirling,” and “wobbling head (head spinning).” For each common TEAE, incidence generally decreased across attacks; findings were similar when the data set included all patients who treated at least 1 attack or the subset who treated all 4 attacks (Table S2).

**Fig. 1:**
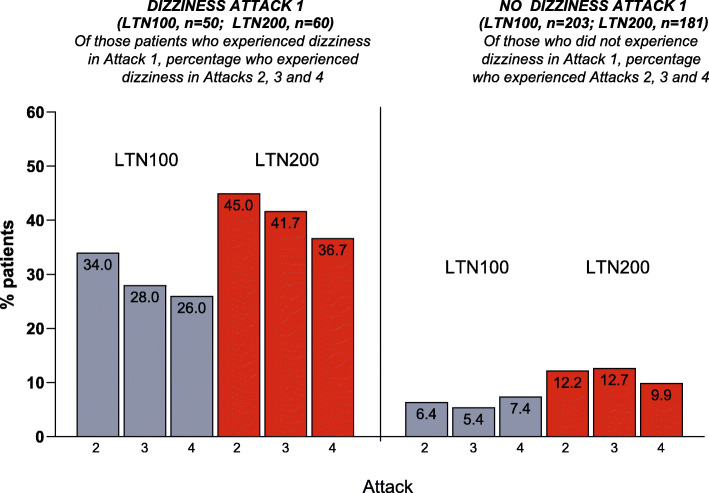
Incidence of dizziness with lasmiditan across attacks in patients who treated 4 attacks

For patients treating all 4 attacks, the subgroup of lasmiditan-treated patients reporting dizziness after treatment of first attack had a lower incidence of dizziness in subsequent attacks (~30 % for lasmiditan 100 mg and 40 % for lasmiditan 200 mg); the subgroup NOT reporting dizziness on treatment of first attack had a low incidence (5-12 %) of dizziness in subsequent attacks (Fig. 1).

Similar findings were observed for other common TEAEs, though numbers were smaller, with 14-44 % of patients who reported a specific common TEAE in the 1st attack also reported the same TEAE in subsequent attacks; patients NOT reporting a common TEAE on treatment of the first attack had a low incidence (<6 %) of that event after treatment of subsequent attacks (Table 4). A decrease in TEAE frequency was also observed in the placebo group (Tables 2 and 3).

**Table 4:** Incidence of common TEAEs with lasmiditan (100 mg and 200 mg dose groups pooled) across attacks in patients who treated 4 attacks

	Reported an event, n (%)				
	**Fatigue**	**Nausea**	**Paresthesia**	**Somnolence**	**Vertigo**
***Reported TEAE in Attack 1***	*N*=494^a^				
Attack 1	37	27	44	29	26
Attack 2	6 (16.2)^b^	12 (44.4)	19 (43.2)	10 (34.5)	9 (34.6)
Attack 3	5 (13.5)	5 (18.5)	19 (43.2)	10 (34.5)	8 (30.8)
Attack 4	7 (18.9)	11 (40.7)	14 (31.8)	5 (17.2)	10 (38.5)
***Did NOT report TEAE in Attack 1***	*N*=457	*N*=467	*N*=450	*N*=465	*N*=468
Attack 1	0	0	0	0	0
Attack 2	12 (2.6)^c^	14 (3.0)	24 (5.3)	8 (1.7)	9 (1.9)
Attack 3	17 (3.7)	11 (2.4)	17 (3.8)	6 (1.3)	8 (1.7)
Attack 4	13 (2.8)	13 (2.8)	18 (4.0)	5 (1.1)	11 (2.4)

^a^Patients in safety population treating all 4 attacks

^b^Denominator is number who reported an event in Attack 1

^c^Denominator is number who did not report an event in Attack 1

#### TEAE severity

**Fig. 2:**
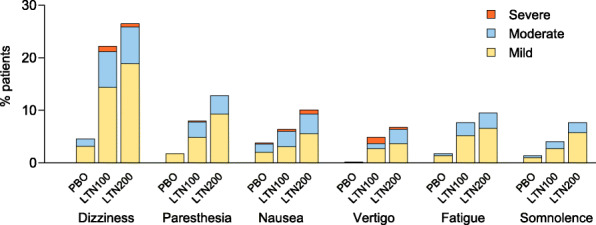
Severity of common TEAEs (Attack 1)

**Fig. 3:**
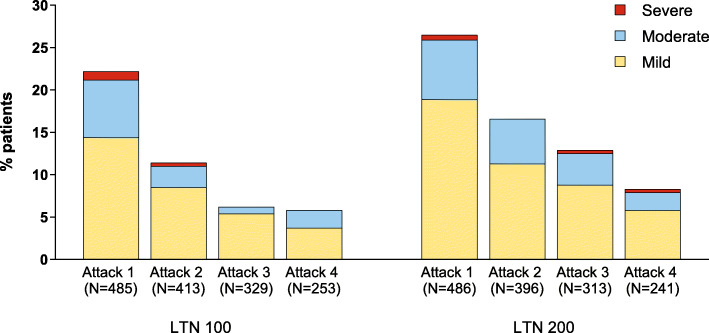
Severity of dizziness by attack in patients who treated ≥ 1 attack

In the first attack, common TEAEs with lasmiditan were generally mild or moderate in severity (Fig. 2). For patients who treated 1 or more attacks (safety population), ≤ 1 % reported severe dizziness with lasmiditan in any individual attack; of those reporting dizziness, <5 % of cases were severe in any attack (Fig. 3). For patients who treated all 4 attacks, ≤ 1 % reported severe dizziness with lasmiditan in any attack. For other common TEAEs, 0-1.2 % of patients reported the event as severe in any attack (maximum percentage reported as severe across attacks were: Fatigue, 0.4 %; nausea, 0.8 %; paresthesia, 0.4 %; somnolence, 0 %; vertigo, 1.2 %).

#### TEAE onset and duration with lasmiditan

For dizziness, time of onset was similar across all treated attacks (~40 min after dosing), as was the duration (~2-3 h) (Table 5).

**Table 5:** Incidence, onset and duration of dizziness with lasmiditan (100 mg and 200 mg doses pooled) by attack

	No. of patients (%)^a^		Onset (median [Q1-Q3]), hrs		Duration (median [Q1-Q3]), hrs	
*Attacks treated*^b^:	*≥ 1*	*All 4*	*≥ 1*	*All 4*	*≥ 1*	*All 4*
Attack 1	212 (21.8)	100 (20.2)	0.7 (0.4-1.2)	0.7 (0.4-1.3)	2.5 (1.0-5.8)	1.8 (1.0-3.9)
Attack 2	124 (15.3)	71 (14.4)	0.7 (0.4-1.0)	0.7 (0.4-1.2)	3.0 (1.2-6.0)	2.0 (1.0-4.5)
Attack 3	87 (13.6)	69 (14.0)	0.5 (0.4-1.0)	0.5 (0.4-1.1)	2.0 (1.0-4.7)	2.0 (1.0-4.0)
Attack 4	62 (12.6)	62 (12.6)	0.7 (0.5-1.0)	0.7 (0.5-1.0)	1.8 (1.0-3.2)	1.8 (1.0-3.2)

^a^Only patients with onset time recorded

^b^Two analyses were performed - (1) with data from patients who treated at least one attack; and (2) with data from patients who treated all 4 attacks

For other common TEAEs, time of onset ranged from ~40 min to 1 h (depending upon the specific TEAE); for each specific TEAE, the onset was similar across attacks (Table S2). The duration of other common TEAEs was variable and dependent upon specific TEAE and attack. It was shortest for paresthesia (less than 2 h for all attacks) and ranged from 1.8 to 5.5 h for other common TEAEs. Apart from fatigue and somnolence, duration was generally similar across attacks (Table S2).

### TEAEs by geography

Overall TEAE incidence was similar across regions for treatment groups. In the first attack, incidences in placebo and lasmiditan pooled (100 mg and 200 mg) were as follows - EU, 23 % and 59 %, respectively; N. America, 21 % and 47 %; Asia, 18 % and 51 %).

The incidence of dizziness was numerically higher in Asia versus EU and N. America. First attack incidences for pooled lasmiditan doses were 32 %, 24 % and 20 %, respectively. For vertigo, the majority of cases with lasmiditan were reported in Europe (136 of the 142 cases across attacks).

### Other safety findings of interest

#### Serotonin syndrome

Serotonin syndrome was reported for 2 patients after taking lasmiditan (Table 2). One case was reported as serious and met Sternbach’s and Hunter’s criteria. The patient had a history of depression and anxiety. Shortly after taking study drug (lasmiditan 200 mg) to treat a migraine attack, the patient experienced CNS symptoms and findings (including myoclonus, ataxia and increased muscle reflexes) along with sweating and increased temperature. Concomitant medications included desogestrel and metoclopramide for migraine prophylaxis, and naratriptan and ibuprofen for acute treatment of attacks. The timing of most recent dose of naratriptan and metoclopramide were not confirmed. The patient was hospitalized and treated with lorazepam 1 mg; symptoms and neurological examination normalized the next day and patient was discharged. There was one non-serious TEAE of serotonin syndrome reported and this case did not meet diagnostic criteria for serotonin syndrome.

#### Suicidality

There were no meaningful differences across treatment groups with regards to suicidal ideation (control group, 1.5 %; lasmiditan 100 mg, 1.1 %; lasmiditan 200 mg, 0.9 %); no patient reported suicidal behavior or self-injury without suicidal intent.

#### Motor vehicle accidents

Patients were instructed not to drive within 8 h of dosing; a driving questionnaire helped to assess whether, in this context, there remained any risk of motor vehicle accidents or driving citations.

Based on responses to a driving questionnaire, motor vehicle accidents with the patient as the driver after taking a dose of study intervention were as follows - one event after placebo, one after lasmiditan 100 mg and one after lasmiditan 200 mg; there were no moving violations/citations after taking study drug.

#### Injuries

Few injuries were reported over the course of the study (8 with lasmiditan, 0 with placebo). The 8 events with lasmiditan (occurring within 48 h of dosing) were limb injury (n=2), epicondylitis, fall, joint dislocation, ligament sprain, muscle rupture and rib fracture; no patient had multiple events. Of the 8 injury-related cases with lasmiditan, 3 patients also reported a central nervous system (CNS) TEAE during the same attack - joint dislocation occurred a few hours after the resolution of formication and vertigo; rib fracture occurred more than a day after the resolution of paresthesia; one case of limb injury occurred approximately one hour after the resolution of dizziness. None of the injuries were considered related to treatment.

## Discussion

In CENTURION, patients treated up to 4 consecutive attacks, permitting assessment of safety over multiple treated attacks.

There were no deaths or major cardiovascular events and lasmiditan was not associated with any increased risk of serious AEs. The most common TEAEs with lasmiditan were dizziness, nausea, paresthesia, fatigue, somnolence and vertigo. In general, the incidences of common TEAEs during the first attack in CENTURION were higher than in previous single-attack Phase 3 studies. In a pooled analysis of data from SAMURAI and SPARTAN (single attack studies), the incidence of any TEAE was 14 % with placebo, 36 % with lasmiditan 100 mg and 41 % with lasmiditan 200 mg compared with 22 %, 53 % and 61 %, respectively in CENTURION (first attack data). In SAMURAI+SPARTAN, the incidence of dizziness was 2.9 % with placebo, 15 % with lasmiditan 100 mg and 17 % with lasmiditan 200 mg ; in CENTURION, first attack incidences of dizziness were 4.6 %, 22 % and 27 %, respectively. Other common TEAEs exhibited similar differences. It is possible that the adverse event data collection methodology or differences in the informed consent documentation [7] in CENTURION may have resulted in a higher incidence of reported TEAEs.

In CENTURION, the incidence of dizziness decreased with repeated dosing and severity findings did not appear to increase across attacks; findings were consistent for the safety population (treated one or more attacks) and for the subgroup who treated all 4 attacks, suggesting that findings were not influenced by drop out of patients who experienced dizziness. For other common TEAEs, incidence also decreased with repeated dosing. The decreases in TEAEs across attacks treated with lasmiditan in the CENTURION study were similar to decreases observed in the GLADIATOR study [8]. In general, severity did not appear to increase across attacks, and duration of all common TEAEs was generally consistent across attacks. We also explored whether reporting dizziness in 1st attack influenced the reporting of the specific event in subsequent attacks. For patients reporting dizziness with lasmiditan treatment during the first attack only 34 to 45 % reported dizziness in any subsequent treated attack; of patients not reporting dizziness on treatment of first attack, few reported dizziness in any subsequent treated attack. Dizziness was generally mild or moderate across attacks. This pattern was similar for other common TEAEs.

Assessment of TEAEs across geographic regions revealed some differences in reporting of dizziness versus vertigo. The majority of vertigo cases were reported in Europe, while the incidence of dizziness was highest in Asia. It is postulated that these differences are the result of cultural or language differences. For example, in German both “Vertigo” and “Dizziness” are captured under the common term “Schwindel” [7]. Other environmental differences or genetic differences across geographic regions cannot be ruled out.

Other safety findings are also reported. Serotonin syndrome was reported for 2 patients after taking lasmiditan; in one patient, this was reported as serious and met Sternbach’s and Hunter’s criteria for the diagnosis of serotonin syndrome. In the CENTURION trial setting, with an 8-hour driving restriction after taking study drug (per protocol), there was no evidence of an increase in motor vehicle accidents. Based on these findings, the risk mitigation strategy of not driving for 8 h appears to be effective and appropriate.

Lasmiditan is centrally penetrant, and was associated with an increased incidence of CNS TEAEs. Of the 8 injury-related TEAEs with lasmiditan, 3 occurred after resolution of a CNS TEAE, and none were considered related to treatment. There was no evidence of any change in suicidality. There were no major ischemic cardiovascular events suggestive of vasoconstriction. These findings are similar to those from SAMURAI and SPARTAN [5].

The strength of the CENTURION study is that it was a large study conducted primarily in Europe with additional sites from North America and Asia; previous lasmiditan phase 3 studies were primarily conducted in the United States. CENTURION also provides lasmiditan safety data collected in a double blind and controlled fashion across multiple attacks.

There are limitations to this study. Direct comparison of TEAE findings for active treatment versus placebo across 4 attacks is not possible; depending on treatment group assignment, patients could treat up to 4 attacks with lasmiditan 100 mg, up to 4 attacks with lasmiditan 200 mg, or up to 3 attacks with placebo (control group). Patients in the control group also received lasmiditan 50 mg for 1 attack and the study was not designed to fully assess the effects of this dose. Since study drug was taken at home when needed and labs/vital signs/ECGs were not collected in temporal proximity to dosing, transient effects may not have been detected. Additional details of particular AEs are unknown, as AEs were generally transient and experienced at home during a migraine attack, and there was no opportunity for the investigator to examine patients during events.

In conclusion, the CENTURION safety findings provide further insight into the safety profile of lasmiditan for patients treating multiple attacks. TEAEs were generally CNS related, mild to moderate in severity, transient in duration, and not associated with injury or other sequelae. The most common TEAEs were similar to those previously reported for Phase 3 single-dose lasmiditan studies; the incidences of TEAEs were somewhat higher than previously reported but decreased over the 4 attacks. For patients who experienced a common TEAE with the first attack, less than half experienced the event in subsequent attacks. Patients who did not report an event in the first attack infrequently reported it in subsequent attacks. Lasmiditan was not associated with major cardiovascular events, with injury, or suicidality. Within the context of a recommendation to not drive for 8 h after dosing, lasmiditan was not associated with motor vehicle accidents.

## Supplementary Information

**Additional file 1.**

## Data Availability

Data are available to request 6 months after the indication studied has been approved in the US and EU. For details on submitting a request, please see the instructions provided at www.clinicalstudydatarequest.com.
