# Supplementary material for: Safety findings from CENTURION, a phase 3 consistency study of lasmiditan for the acute treatment of migraine
Source: J Headache Pain. 2021 Nov 6;22(1):132. doi: 10.1186/s10194-021-01343-2 (PMC8572440; doi:10.1186/s10194-021-01343-2)
Supplement: Supplementary file 1 — Additional file 1. [file 10194_2021_1343_MOESM1_ESM.docx]

# SUPPLEMENT

## List of ERBs for the CENTURION study

| Advarra Inc.  6940 Columbia Gateway Drive IRB Suite 110  Columbia, MD, 21046, US |
| --- |
| Quorum Review Inc.  1501 Fourth Avenue Suite 800  Seattle, WA, 98101, US |
| Georgetown University Hospital  SW 104 Med-Dent Bldg  3900 Reservori Rd, NW Ste SW104  Washington, DC, 20057, US |
| Western Institutional Review Board-WIRB  1019 39^th^ Avenue SE Suite 120  Puyallup, WA, 98374-2115, US |
| Biomedical Research Alliance of New York  1981 Marcus Ave Suite 210  Lake Success, NY, 11042, US |
| Landesamt für Gesundheit und Soziales (LAGeSo)  Geschäftsstelle der Ethik-Kommission des Landes  Berlin, Fehrbelliner Platz 1  Berlin, Berlin, 10707, DE |
| Instituto de Investigaciones Aplicadas a la Neurociencia A.C  Pedro Moreno, 934 Col Centro  Guadalajara, 44100, MX |
| Comite de Etica en investigacion del Sanatorio Alcocer Pozo  Reforma 23, Col. Centro  Querétaro, Querétaro, 76000, MX |
| Medical Care and Research, S.A. de C.V.  Calle 32 Num. 217 x 11-A Col. Garcia Gineres  Merida, Yucatan, 97070, MX |
| Hospital la Misión  Avenida del Hospital 112, 1º y 2º piso, Sertoma  Monterrey, Nuevo Leon, 64718, MX |
| Comite de Etica en Investigacion de Chirurgie & Medical  Darwin número 107, Colonia Anzures,  Miguel Hidalgo, Federal District, 11590, MX |
| Deenanth Mangeshkar Hospital and Research Centre  Multi Specialty Building, Erandawane  Pune, Maharashtra, 411004, IN |
| Artemis Hospital  Sector - 51  Gurgaon, Haryana, 122001, IN |
| Nizam's Institute of Medical Sciences  Punjagutta  Hyderabad, Andhra Pradesh, 500082, IN |
| Gobind Ballabh Pant Hospital  Room no 504, Academic block GIPMER Jawaharlal Nehru Marg  New Delhi, 110002, IN |
| Sir Ganga Ram Hospital  Rajinder Nagar  New Delhi, Delhi, 110060, IN |
| M S Ramaiah Medical College Hospital  First Floor, M S Ramaiah Advanced Learning Centre M S R Nagar, MSRIT Post,  Bangalore, Karnataka, 560054, IN |
| Mangala Hospitals & Mangala Kidney Foundation  Room No: 604, 6th Floor, Vajra Hills Kadri Road  Mangalore, Karnataka, 575003, IN |
| HCG Manavata Cancer Centre  Behind Shivang Auto, Mumbai Naka  Nasik, Maharashtra, 422001, IN |
| EK der Medizinischen Universität Wien  Borschkegasse 8B/E06  Wien, Wien, 1090, AT |
| Universitair Ziekenhuis Gent  Corneel Heymanslaan 10, Ingang 75 route 7522 Commissie voor Medische Ethiek  Gent, 9000, BE |
| Bimetra - UZ Gent  C. Heymanslaan 10 Ingang 81 \| Route 812  Gent, 9000, BE |
| Institute klinicke a experimentalni mediciny - IKEM  Videnska 800 Thomayerovy nemocnice  Praha, Praha 4 – Krc, 140 59, CZ |
| Eticka komise NZZ CLINTRIAL, s.r.o  Pocernicka 1427/16  Praha 10, 100 00, CZ |
| De Videnskabsetiske Komiteer for Region Hovedstaden  Regionsgaarden, Kongens Vaenge 2  Hilleroed, 3400, DK |
| Chu de Grenoble  Comité de Protection des Personnes Sud Est V 6 Bd de la Chantourne  La Tronche, 38700, FR |
| Egeszsegugyi Tudomanyos Tanacs  Szechenyi Istvan ter 7-8 KFEB  Budapest, 1051, HU |
| Istituto Neurologico Neuromed  Località Camerelle -Santa Maria Oliveto Via Atinense 18  Pozzilli, Isernia, 86070, IT |
| Ospedale Bellaria  Segreteria Locale Via Castiglione, 29  Bologna, Bologna, 40100, IT |
|  |
| Isala Klinieken  Dokter van Deenweg 1 Gebouw M (Mondriaan), kamer 0.25, Postbus  Zwolle, 10400, NL |
| Hospital Universitario Virgen del Rocio  Avenida Manuel Siurot s/n  CEI Hosp.Univ.V.Macarena,V.Rocio-Edif. de Lab plta 6ª  Sevilla, Andalucía, 41013, ES |
|  |
| CEI Hosp. Univ. V. Macarena, V. Rocio-Edif. de Lab plta 6ª, Avenida Manuel Siurot s/n  Sevilla, Andalucía, 41013, ES |
| Ethikkommission Nordwest- und Zentralschweiz (EKNZ)  Hebelstrasse 53  Basel, Basel Stadt, 4056, CH |
| Riverside Research Ethics Committee  Level 3 Block B Whitefriars  Lewins Mead  Bristol, London, BS1 2NT, GB |
| Saint Petersburg State Medical University n.a. Pavlov I.P.  UI. L.Tolstogo 6/8  Saint Petersburg, 197022, RU |
| First Moscow State Medical University n.a. Sechenov  Trubetskaya street 8, building 2  Moscow, 119991, RU |
| University Headache Clinic  Molodogvardeiskaia street, 2 building 1  Moscow,121467, RU |
| Medis Priokskiy  Tropinina Street 4a  Nizhny Novgorod, 603137, RU |
| Affiliated Hospital of Jiangsu University  4F, Medical Building,No.438, Jiefang Road  Zhenjiang, Jiangsu, 212001, CN |
| The First Affliated Hospital of Suzhou University  Room 403, Science and Teaching Building No.188 Shizi Street  Suzhou, Jiangsu, 215006, CN |
| The Second Affiliated Hospital of Zhejiang University School of Medicine  Room 1601, Out-patient Building No.88 Jiefang Road  Hangzhou, Zhejiang, 310009, CN |
| The First Affiliated Hospital Chongqing Medical University  Room 1103, No. 5 Building No.1.Youyi Rd, Yuanjia Gang  Chongqing, Chongqing, 400016, CN |
| First Hospital affiliated to Zhengzhou University  ERB office, 2nd Floor, Building 15B No. 50 Jianshe East Road, Erqi District  Zhengzhou, Henan, 450052, CN |
| Hebei General Hospital  Room 623, 6th Floor Outpatient Building No. 348 Heping West Road  ShiJiazhuang, Hebei, 050051, CN |
| HuaShan Hospital Affiliated To Fudan University  No. 12 Wulumuqi Zhong Road, Jingan District  Shanghai, Shanghai, 20040, CN |
| No.2 Hospital Affiliated to Jilin University  No 218, Ziqiang Street, Nanguan District  Changchun City, Jilin, 130041, CN |
| Chinese PLA General Hospital  No. 28 Fu Xing Road, Haidian District  Beijing, 100853, CN |
| Guangzhou First People's Hospital  No.1 Panfu Road, Yuexiu district  Guangzhou, Guangdong, 510180, CN |
| Xiangya Hospital, Central South University  209 room, 2^nd^ floor, Pharmacy building, 87 Xiangya Road  Changsha, Hunan, 410008, CN |
| First Affiliated Hospital of Xi'an Jiaotong University  No.277 Yanta West Rd.  Xi'an, Shaanxi, 710061, CN |
| Wuhan Union Hospital  1612 Ethics Comm Office 2 Building, Foundation Med College  Wuhan, Hubei, 430022, CN |
| West China Hospital of Sichuan University  No. 37 Guoxue Lane Room 412, 4th floor, 8th teaching building  Chengdu, Sichuan, 610041, CN |
| First Affiliated Hospital of Kunming Medical University  EC office, 3rd Floor, Administration Building No.2 Kunshi Rd  Kunming, Yunnan, 650221, CN |
| People's Hospital of Rizhao  The 4th floor, 8th building No.126 Taian road, Donggang District  Rizhao, ShanDong, 276826, CN |
| Baotou Central Hospital  ERB Office, 2F Out-patient Building NO.61 Huancheng Road, Donghe District  Baotou, Neimenggu Zizhiqu, 014040, CN |
| Pingxiang People's Hospital  ERB Office, 2nd Floor, Nuclear magnetic resonance building No. 8, Wugongshan Avenue  Piang xiang, Jiangxi, 337055, CN |
| The First Affiliated Hospital of Wenzhou Medical College  A07 Room , 4th Floor, NO 1 Building, New Campus Nanbaixiang Wenyi First Hospital, Ouhai District  WenZhou, ZheJiang, 325000, CN |
| Dalian Municipal Central Hospital Affiliated of Dalian Medical University  3F, the office building, southern branch Medical University, No.42 Xuegong Street, Shahekou District  Dalian, Liaoning, 116033, CN |
| Tianjin Medical University General Hospital  The Ethics Committee, Drug Clinical Office, 1st Floor,  Research Building, No. 154 Anshan Road, Heping District  Tianjin, 300052, CN |
| Xuanwu Hospital-Capital Medical University  Room 301, the fifth complex building No. 45 Changchun Street, Xicheng district  Beijing, 100053, CN |

## Table S1 - Summary of study drug exposure for the safety population during the double-blind randomized treatment period

|  | | **Control** | **Lasmiditan 100mg** | **Lasmiditan 200mg** | **Total** |
| --- | --- | --- | --- | --- | --- |
| **Number randomized** | | 538 | 539 | 536 | 1613 |
| **Number who treated an attack** | | 500 (92.9) | 485 (90.0) | 486 (90.7) | 1471 (91.2) |
| **Attacks treated** | **0** | 38 (7.1) | 54 (10.0) | 50 (9.3) | 142 (8.8) |
|  | **1** | 62 (12.4) | 72 (14.8) | 90 (18.5) | 224 (15.2) |
|  | **2** | 64 (12.8) | 84 (17.3) | 83 (17.1) | 231 (15.7) |
|  | **3** | 108 (21.6) | 76 (15.7) | 72 (14.8) | 256 (17.4) |
|  | **≥4^a^** | 266 (53.2) | 253 (52.2) | 241 (49.6) | 760 (51.7) |

^a^All patients received intervention to treat 4 migraine attacks, but 35 patients also treated attacks with non-study medication; these patients treated 5-8 attacks in total during the double blind period

## Table S2 - Incidence, onset and duration of common TEAEs with lasmiditan (100 mg and 200 mg dose groups pooled) by attack

|  | **n (%)^a^** | | **Onset** | | **Duration** | |
| --- | --- | --- | --- | --- | --- | --- |
| *Attacks treated:* | *≥1* | *All 4* | *≥1* | *All 4* | *≥1* | *All 4* |
| **Dizziness** | | | | | | |
| Attack 1 | 212 (21.8) | 100 (20.2) | 0.7 (0.4-1.2) | 0.7 (0.4-1.3) | 2.5 (1.0-5.8) | 1.8 (1.0-3.9) |
| Attack 2 | 124 (15.3) | 71 (14.4) | 0.7 (0.4-1.0) | 0.7 (0.4-1.2) | 3.0 (1.2-6.0) | 2.0 (1.0-4.5) |
| Attack 3 | 87 (13.6) | 69 (14.0) | 0.5 (0.4-1.0) | 0.5 (0.4-1.1) | 2.0 (1.0-4.7) | 2.0 (1.0-4.0) |
| Attack 4 | 62 (12.6) | 62 (12.6) | 0.7 (0.5-1.0) | 0.7 (0.5-1.0) | 1.8 (1.0-3.2) | 1.8 (1.0-3.2) |
| **Paresthesia** | | | | | | |
| Attack 1 | 93 (9.6) | 40 (8.1) | 0.7 (0.4-1.0) | 0.7 (0.3-0.9) | 1.8 (0.6-3.5) | 1.3 (0.6-2.3) |
| Attack 2 | 73 (9.0) | 41 (8.3) | 0.6 (0.4-1.0) | 0.6 (0.5-0.9) | 1.8 (0.7-4.0) | 1.8 (0.7-3.5) |
| Attack 3 | 49 (7.6) | 35 (7.1) | 0.6 (0.3-1.0) | 0.6 (0.3-1.0) | 1.1 (0.5-2.2) | 1.0 (0.5-2.0) |
| Attack 4 | 31 (6.3) | 31 (6.3) | 0.6 (0.5-1.1) | 0.6 (0.5-1.1) | 1.2 (0.5-2.5) | 1.2 (0.5-2.5) |
| **Fatigue** | | | | | | |
| Attack 1 | 76 (7.8) | 35 (7.1) | 0.9 (0.6-1.8) | 1.2 (0.6-2.2) | 4.0 (2.0-9.0) | 3.0 (2.0-8.5) |
| Attack 2 | 40 (4.9) | 17 (3.4) | 0.9 (0.4-1.4) | 0.9 (0.5-1.5) | 5.5 (2.5-10.0) | 2.8 (2.0-5.0) |
| Attack 3 | 27 (4.2) | 20 (4.0) | 0.8 (0.5-2.3) | 0.7 (0.5-1.3) | 5.0 (2.0-9.7) | 5.2 (1.5-9.7) |
| Attack 4 | 19 (3.8) | 19 (3.8) | 1.0 (0.6-2.0) | 1.0 (0.6-2.0) | 5.0 (2.5-9.5) | 5.0 (2.5-9.5) |
| **Nausea** | | | | | | |
| Attack 1 | 70 (7.2) | 22 (4.5) | 1.0 (0.3-2.0) | 1.3 (0.3-4.0) | 4.8 (2.0-9.0) | 3.3 (1.5-7.7) |
| Attack 2 | 38 (4.7) | 24 (4.9) | 0.9 (0.5-1.7) | 0.9 (0.5-1.9) | 3.9 (2.0-8.1) | 2.8 (1.6-6.0) |
| Attack 3 | 20 (3.1) | 15 (3.0) | 0.9 (0.5-3.1) | 0.7 (0.4-2.0) | 2.9 (2.0-10.8) | 2.0 (2.0-5.3) |
| Attack 4 | 23 (4.7) | 23 (4.7) | 0.8 (0.5-1.4) | 0.8 (0.5-1.4) | 3.0 (2.0-6.3) | 3.0 (2.0-6.3) |
| **Vertigo** | | | | | | |
| Attack 1 | 54 (5.6) | 23 (4.7) | 0.7 (0.4-1.2) | 1.1 (0.4-3.0) | 5.0 (1.9-8.9) | 3.7 (2.0-9.0) |
| Attack 2 | 36 (4.4) | 16 (3.2) | 0.7 (0.4-1.0) | 0.5 (0.3-0.8) | 3.0 (1.3-5.0) | 3.2 (1.4-5.0) |
| Attack 3 | 26 (4.0) | 14 (2.8) | 1.0 (0.5-2.3) | 0.9 (0.5-2.0) | 2.4 (1.2-5.0) | 1.8 (1.0-3.0) |
| Attack 4 | 18 (3.6) | 18 (3.6) | 0.9 (0.6-2.0) | 0.9 (0.6-2.0) | 2.5 (1.0-3.7) | 2.5 (1.0-3.7) |
| **Somnolence** | | | | | | |
| Attack 1 | 47 (4.8) | 25 (5.1) | 1.0 (0.5-1.8) | 1.0 (0.5-1.6) | 3.8 (2.0-8.0) | 2.6 (1.2-7.9) |
| Attack 2 | 37 (4.6) | 17 (3.4) | 0.8 (0.5-1.5) | 0.8 (0.6-1.5) | 4.3 (2.0-10.3) | 4.8 (2.3-10.6) |
| Attack 3 | 22 (3.4) | 14 (2.8) | 0.9 (0.5-1.5) | 0.9 (0.5-1.9) | 3.0 (2.0-5.0) | 2.0 (2.0-8.0) |
| Attack 4 | 10 (2.0) | 10 (2.0) | 0.6 (0.3-0.9) | 0.6 (0.3-0.9) | 5.2 (2.0-24.0) | 5.2 (2.0-24.0) |

^a^only patients with onset time recorded
